# Epigenetic Alterations in Renal Cell Cancer With TKIs Resistance: From Mechanisms to Clinical Applications

**DOI:** 10.3389/fgene.2020.562868

**Published:** 2021-01-12

**Authors:** Qinhan Li, Zhenan Zhang, Yu Fan, Qian Zhang

**Affiliations:** Department of Urology, Peking University First Hospital, Institute of Urology, National Research Center for Genitourinary Oncology, Peking University, Beijing, China

**Keywords:** renal cell carcinoma, epigenetics, microRNA, long non-coding RNA, methylation, histone modification, target therapy, tyrosine kinase inhibitor

## Abstract

The appearance of tyrosine kinase inhibitors (TKIs) has been a major breakthrough in renal cell carcinoma (RCC) therapy. Unfortunately, a portion of patients with TKIs resistance experience disease progression after TKIs therapy. Epigenetic alterations play an important role in the development of TKIs resistance. Current evidence suggests that epigenetic alterations occur frequently in RCC patients with poor response to TKIs therapy, and modulation of them could enhance the cytotoxic effect of antitumor therapy. In this review, we summarize the currently known epigenetic alterations relating to TKIs resistance in RCC, focusing on DNA methylation, non-coding RNAs (ncRNAs), histone modifications, and their interactions with TKIs treatment. In addition, we discuss application of epigenetic alteration analyses in the clinical setting to predict prognosis of patients with TKIs treatment, and the potential use of epigenetics-based therapies to surmount TKIs resistance.

## Introduction

Renal cell carcinoma (RCC) is the most common type of renal cancer, causing more than 14,000 deaths yearly (Capitanio et al., 2019). For early stage of RCC, surgical excision is the recommended treatment. However, there are nearly 15% of patients with distant metastasis when diagnosed with RCC (Siegel et al., 2019).

Angiogenesis plays an important role in the biology and the pathogenesis of RCC. Loss of function of von Hippel–Lindau (VHL) tumor suppressor gene is a vital event in renal carcinogenesis and occurs in about 90% of all clear cell renal cell cancer (ccRCC; Nickerson et al., 2008). VHL encodes and forms a VHL protein complex, which acts as an essential factor in the oxygen-sensing pathway through ubiquitin-mediated degradation of hydroxylated hypoxia inducible factor 1 (HIF-1*α*) and HIF-2α (Maxwell et al., 1999; Kaelin, 2002). Loss of VHL function leads to the accumulation of HIF-1α and HIF-2α, which consequently facilitates transcription of the hypoxia response genes, such as genes in vascular endothelial growth factor (VEGF), platelet-derived growth factor (PDGF), and transforming growth factor alpha (TGF-*α*), eventually, resulting in angiogenesis and progression of tumor (Kourembanas et al., 1990; de Paulsen et al., 2001). The expression of VEGF and PDGF is significantly upregulated in RCC as a result of VHL inactivation, which, on the one hand, accelerates growth of tumor, on the other hand, is also its weakness. Tyrosine kinase inhibitors (TKIs), including sunitinib, pazopanib, axitinib, sorafenib, and cabozanitinib are thought to exert their major therapeutic effects in RCC by antagonism of VEGF receptor (VEGFR) and PDGF receptor (PDGFR), leading to a reduction of tumor angiogenesis.

For metastatic RCC (mRCC), sunitinib, pazopanib, and cabozantinib are approved for first-line treatment, while axitinib and sorafenib are chosen as second-line treatment. Sunitinib is the most commonly used TKIs which can delay tumor progression and improve patient survival. However, only 20–30% of patients respond to sunitinib treatment initially, and almost all initial responders develop resistance in 2 years (Morais, 2014). Subsequent antitumor therapies are followed by immune-checkpoint inhibitor and mammalian target of rapamycin (mTOR), such as nivolumab and everolimus. TKIs resistance poses a great challenge for the TKIs treatment. Therefore, understanding the distinct molecular mechanisms underlying TKIs resistance is vital to find efficient biomarkers to predict the effect of TKIs and facilitate the development of novel antitumor drugs which overcome this resistance.

## An Overview of Epigenetic Modification

Epigenetics refers to the study of molecules and mechanisms that can control chromatin structure and influence gene expression or the propensity for genes to be transcribed within organisms in the context of the same DNA sequence. The ability of cells to retain and transmit their special gene expression patterns to the progeny cells, referred to as epigenetic memory, is governed by epigenetic marks, such as DNA methylations, histone modifications, and non-coding RNAs (ncRNAs; Thiagalingam, 2020). Epigenetic modification is heritable but reversible (Cavalli and Heard, 2019). The unique epigenome defining the genetic code associated with each individual gene regulates the expression status of that gene. Defects in epigenetic factors and epigenetic modifications could act as pushers for various diseases including cancer.

Epigenetic modification is associated with drug resistance in numerous types of cancer, including RCC (Chekhun et al., 2007; Knoechel et al., 2014; Adelaiye-Ogala et al., 2017; Leonetti et al., 2019), which regulates gene expression at the protein level (histone modification and nucleosome remodeling), DNA level (DNA methylation), and RNA level (ncRNA). Histones are the central component of nucleosomal subunit, including four types of histone proteins [histone 2A (H2A), H2B, H3, and H4], which are wrapped by a 147-base-pair segment of DNA (Santos-Rosa and Caldas, 2005; Audia and Campbell, 2016). Histone modifications mainly take place at histone tails, which are densely populated with basic lysine and arginine residues (Audia and Campbell, 2016). The acetylation and methylation of lysine residues are well-known. Acetylation can alter the charge on the lysine residues and weaken the interaction of these histones with DNA, making the chromatin structure more open and accessible (Dawson and Kouzarides, 2012). This process is regulated by two enzymatic families with competing activities: promoted by histone lysine acetyltransferases (HATs) and inhibited by the histone deacetylases (HDACs; Li et al., 2019). Methylation of lysine residues in histone tails contains three forms: monomethylation (me1), demethylation (me2), and trimethylation (me3), making activation or repression of transcription (Kouzarides, 2007). This process is also competitively regulated by histone lysine methyltransferases (KMTs) and histone lysine demethylases (KDMs). Generally, acetylation of lysine 14 of H3 (H3K14), monmethylation of H3K4, H3K9, and H3K79, and phosphorylation of serine 10 (H3S10) are all linked with transcriptional activity (Cheung et al., 2000; Lo et al., 2000; Barski et al., 2007), while trimethylation of H3K9, H3K79, and H3K27 marks transcriptional repression (Boyer et al., 2006; Barski et al., 2007).

At the DNA level, the methylation of the 5-carbon on cytosine CpG dinucleotides is considered as an important epigenetic marker. Catalyzed by DNA methyltransferases (DNMTs), 5-carbon of the cytosine ring on promoter CpG islands gets a methyl from S-adenosylmethionine, converting to 5-methycytosine (5mc). 5mc attracts HDACs and methy-CpG-binding domain proteins (MBDs) to the site, resulting in removal of acetyl groups from histone proteins, compact conformation of nucleosome, and downregulation of gene transcription (Robert et al., 2003; Feinberg and Tycko, 2004; Wang et al., 2009). This process can be reversed by ten-eleven translocation (TET) proteins, which oxidize 5mc into 5-hydroxymethylcytosine (5hmc) and subsequently into 5-formylcytosine (5fC) and 5-carboxylcytosine (5caC) in an Fe(II)- and 2-oxoglutarate-dependent dioxygenases manner (Wu and Zhang, 2014; Rasmussen and Helin, 2016).

Non-coding RNAs regulate gene expression at RNA level, including mircoRNAs (miRNAs), small nucleolar RNAs (snoRNAs), piwiRNAs (piRNAs), and long ncRNA (lncRNA; Ma et al., 2013). In general, composed by about 19–25 nucleotides, miRNAs can lead to posttranscriptional gene silencing and translation stopping through binding to the 3'-untranslated region (3'-UTR) of the targeted messenger RNAs (mRNAs) and leading to its degradation or destabilization (Brennecke et al., 2005). lncRNAs are collectively defined as longer than 200 nucleotides in length, which modulate local or global gene expression in a neighboring (*cis*) or distal (*trans*) manner (Kopp and Mendell, 2018). For example, one classic *cis*-acting lncRNA is the X-inactive specific transcript (Xist) resulting in the X chromosome inaction (XCI) in mammals by recruiting various protein complexes to specific position (Lee and Bartolomei, 2013). Notably, lncRNAs can function as competing endogenous RNAs (ceRNAs) to compete with miRNAs by binding to their protein-coding transcripts, thereby antagonizing the repressive effects of miRNAs on mRNAs (Salmena et al., 2011; Du et al., 2016).

In this review, we summarize the currently known epigenetic alterations relating to TKIs resistance in RCC, focusing on DNA methylation, ncRNAs, histone modifications, and their interactions with TKIs treatment. In addition, we discuss the application of epigenetic alteration analyses in the clinical setting to predict prognosis of patients with TKIs treatment and develop new agents.

### Mechanisms of Primary and Acquired Resistance to TKIs Treatment

There is no specific definition of TKIs resistance in RCC. Response to drug therapy is normally defined by the Response Evaluation Criteria In Solid Tumors (RECIST) criteria as evidence of tumor progression regardless of persistent treatment. Unfortunately, the current clinical studies depended on their own criteria to divide patients into responders and non-responders, which made their outcomes difficult to compare. Resistance to antiangiogenic therapy can be classified into intrinsic (primary) and acquired (secondary) resistance (Mollica et al., 2019). Intrinsic resistance is defined as an initial inefficacy of therapeutic agents, which may be attributed to the presence of resistant tumor clones prior to therapy due to inherited resistance or evolutionary clonal selection. Acquired resistance is classified as the progression of tumor after initial tumor regression during the therapy, which is often driven by the development of other pathways stimulating angiogenesis, such as AXL, MET, and PDGF/PDGFR, and thus the escape of cancer cells from VEGF/VEGFR blockade (Crawford et al., 2009; Zhou et al., 2016). While the explicit mechanisms of TKIs resistance are still being explored, several potential factors have been reported to be associated with TKIs resistance in RCC: lysosomal sequestration, mutations and modification of expression level, downstream signaling pathway activation, bypass or alternative pathway activation, ATP-binding cassette (ABC) efflux transporters, tumor microenvironment, epithelial-mesenchymal transition (EMT), and epigenetic modification (Housman et al., 2014; Makhov et al., 2018). Epigenetic regulation of the TKIs resistance is always linked to activation of downstream signaling pathways, promotion of EMT, and stimulation of alternative pathways.

#### Modulation of Downstream Signaling Pathways

Tyrosine kinase inhibitors exert their major antiangiogenic and antitumor effect in RCC by suppressing tyrosine kinase receptors on VEGFR and PDGFR and inhibiting their downstream signaling pathways. Therefore, RCC cells escape TKIs blockade through an important mechanism of activation of parallel downstream signaling pathways, among which PI3K/AKT and RAS/RAF/ERK are pivotal transduction cascades responsible for cell survival, proliferation, and invasion (Fresno Vara et al., 2004; Guo et al., 2015; Huang and Fu, 2015). The PI3K/AKT pathway is frequently activated in cancer and leads to the development and progression of numerous tumor types, including RCC (Samuels et al., 2004; Lawrence et al., 2014). PI3K, a family of lipid kinases, is normally activated by extracellular signals, such as growth factors, cytokines in physiologic conditions. Activated PI3K phosphorylates phosphatidylinositol 4,5-bisphosphate [Ptdlns(4,5)P2], propagating activation signals to downstream molecules (Hennessy et al., 2005). Phosphatase and tensin homolog deleted on chromosome 10 (PTEN) can turn off this pathway by inhibiting the phosphorylation of Ptdlns(4,5)P2 (Gewinner et al., 2009). AKT is the key mediator to respond to the PI3K signaling. The phosphorylated active AKT translocates from the cell membrane to other cell compartments to phosphorylate multiple downstream substrates, resulting in cell survival, growth, tumorigenesis, metastasis, and sunitinib resistance (Andjelković et al., 1997; Sakai et al., 2013; Fang et al., 2019). Activated by pAKT, mTOR complex 1 can lead to protein translation and lipid or nucleotide synthesis *via* phosphorylating numerous substrates, such as p70 ribosomal S6 kinase (p70S6K) and Eif4e-binding proteins (Manning and Cantley, 2003; Fruman and Rommel, 2014), eventually leading to the translation and accumulation of HIF-1*α* and HIF-2α. Acting as an inhibitory protein of the pathway, PTEN contributes to the downregulation of AKT activity, and loss of PTEN leads to sunitinib resistance due to lack of inhibitory input (Makhov et al., 2012). Sekino et al. (2019) identified miR-130 upregulation was associated with sunitinib resistance through suppression of PTEN.

Focal adhesion kinase (FAK) signaling plays an important role in activation of PI3K/AKT pathway by interacting with PI3K (Zhao and Guan, 2009; Poettler et al., 2013; Hung et al., 2017). Activation of FAK signaling contributes to the sorafenib and sunitinib resistance in a variety type of cancer, including RCC (Bai et al., 2012; Zhang et al., 2016; Zhou et al., 2017). The chromatin modifier enhancer of zeste homolog 2 (EZH2), a polycomb group protein homolog of Drosophila enhancer of zeste, is a histone methytransferase unit of polycomb repressive (PRC2), which can catalyze the trimethylation of H3K27, change chromatin configuration, and promote transcriptional silencing (Margueron and Reinberg, 2011; Di Croce and Helin, 2013). Adelaiye-Ogala et al. (2017) reported that increased EZH2 was associated with sunitinib resistance through redistribution in RCC cells, decreasingly binding to the *PTK2* gene, which encodes the FAK, and increasingly binding to *DAB2IP* and *PTPN2*, which act as tumor suppressors to inhibit RAS/RAF/ERK and P13K/AKT signaling pathways.

Ras/Raf/ERK signaling pathway is other important transduction cascade transmitting EGFR signaling, responsible for cancer development, maintenance, progression and thus, poorer prognosis and TKIs resistance (Bridgeman et al., 2016; Mandal et al., 2016). The methylation of *glutaminyl peptide cyclotransferase* (*QPCT*) gene had been reported to associate with sunitinib resistance through Ras/Raf/ERK signaling pathway (Zhao et al., 2019). The *QPCT* gene encodes glutaminyl cyclase (QC), an enzyme that is involved in the posttranslational modification by converting the N-terminal glutaminyl and glutamyl into pyroglutamate through cyclization, making the protein more resistant to protease degradation, more hydrophobic, and more prone to aggregation and neurotoxicity (Khan et al., 2016; Vijayan and Zhang, 2019). Hypomethylated *QPCT* gene increased the expression of QC, the process promoted by the NF-*κ*B signaling (p65; Kehlen et al., 2013), leading to upregulation of HRAS and activation of the Ras/Raf/ERK signaling pathway (Herrero et al., 2016; Michael et al., 2016; Zhao et al., 2019). Zhai et al. (2017) had observed that lncRNA-SARCC could regulate androgen receptor (AR) to increase miR-143-3p expression and inhibit its downstream signals, including AKT, MMP-13, K-RAS, and P-ERK. The expression of lncRNA-SARCC was upregulated in RCC cells treated with sunitinib, which was associated with decreased resistance to sunitinib.

### Modulation of Epithelial-to-Mesenchymal Transition

Epithelial-to-mesenchymal transition is a biologic process that epithelial cells lose their cell–cell basement membrane contacts and their structural polarity to become spindle-shaped and morphologically similar to mesenchymal cell (He and Magi-Galluzzi, 2014). While potential mechanisms are not fully explicit, numerous studies indicate that EMT constitutes a relevant resistance mechanism to TKI treatment (Fang et al., 2019; Hwang et al., 2019; Zhu et al., 2019), and relates to the development of metastases in cancer (Bastid, 2012). Signal transduction affects EMT through the TGF-beta 1 (TGF-β1) in different mechanisms (Wendt et al., 2009; Feldkoren et al., 2017; Fardi et al., 2019). Schematically, TGF-β1 activates zinc finger E-box binding 1 (ZEB1) and ZEB2, which are responsible for a key transcriptional repressor of the cadherin 1 gene (CDH1). CDH1 encodes the cell-adhesion glycoprotein E-cadherin whose downregulation is a pivotal hallmark of EMT (Loh et al., 2019). As an activator of EMT, the expression of ZEB2 is regulated by miR-141 (Berkers et al., 2013). In detail, miR-141 downregulation induces EMT and hypoxia resistance through the upregulation of ZEB2 and suppression of E-cadherin, resulting in an unfavorable response to sunitinib resistance and poor prognosis (Berkers et al., 2013; Fang et al., 2013).

The overexpression of EZH2 is beneficial to EMT by repression of E-cadherin (Crea et al., 2012; Liu et al., 2016). Adelaiye-Ogala et al. (2017) reported that EZH2 expression was linked to sunitinib resistance in RCC through an adaptive kinome reprograming, such as increased global tyrosine and serine phosphorylation as well as increased phosphorylated FAK. SOX5, one of SOX family involving in the regulation of tumor progression, is thought to contribute to EMT in different types of cancer (Grimm et al., 2019). Liu et al. (2019) reported lncRNA-GAS5 was responsible to sorafenib resistance by functioning as ceRNA to repress miR-21, which controlled its downstream target SOX5.

The Wnt/β-catenin pathway acts as one of the signaling pathways controlling EMT through directly or indirectly targeting several key transcription factors regulating E-cadherin expression and/or the fate of other epithelial molecules (Valenta et al., 2012). SET and MYND domain-containing protein 2 (SMYD2), which acts as one of the SMYD-methyltransferase protein family and specifically methylates H3K4 through its SET domain (Abu-Farha et al., 2008), is deemed to regulate the expression of miR-125b (Yan et al., 2019). miR-125b bind directly to the 3'-UTR of DKK3, a key regulatory factor in the Wnt/β-catenin pathway which acts as a tumor suppressor in RCC (Lu et al., 2017). Thus, the activation of SMYD2/miRNA-EMT pathway weakens the effect sunitinib treatment and accelerates the tumor growth (Yan et al., 2019).

#### Activation of Bypass Pathways

Extra activation of bypass pathways driving angiogenesis is also one of the most important processes driving TKIs resistance. The activation of MET and AXL confers to the stimulation of their downstream signal cascades, including PI3K and RAS signaling pathway, resulting in sunitinib resistance (Huang and Fu, 2015; Zhou et al., 2016). lncRNA Activated in RCC with Sunitinib Resistance (lncARSR) functions as a sponge and competes for binding of miR-34 and miR-449 to their transcripts, leading to the upregulation of AXL/MET and the activation of STAT3, AKT, and ERK signaling (Qu et al., 2016). miR-32-5p can increase the efficacy of sunitinib by suppressing the testicular nuclear receptor 4 (TR4), which plays an important role in activation of HGF/MET signaling pathway (Wang et al., 2018).

Inactivation of VHL leads to increased HIF-1*α* and HIF-2α. In renal carcinogenesis, HIF-1α functions more as a tumor suppressor than a tumor promoter, whereas HIF-2α is deemed to predominantly promote tumor growth and angiogenesis (Raval et al., 2005). Specifically, HIF-1α inhibits interaction of MYC with its DNA-binding partners by displacing the SP1 transcription factor from MYC, while HIF-2α could enhance MYC activity by forming a complex with MAX, and thus stabilizing the MYC-MAX and MYC-MAX-SP1 complexes (Keith et al., 2011). HIF-2α/C-MYC axis relates to progression and TKIs resistance in RCC (Zhai et al., 2016; Maroto et al., 2017). Beuselinck et al. (2015) segregated specific groups of patients with ccRCC, who presented sunitinib resistance into four molecular tumor subtypes based on their mRNA expression data: ccrcc1 (c-myc-up), ccrcc2 (classical), ccrcc3 (normal-like), and ccrcc4 (c-myc-up and immune-up). ccrcc1/ccrcc4 subtypes posed a hypomethylation of *MYC* gene and a global hypermethylation level, with overexpression of *MYC* and down-expression of corresponding genes, such as *PRC2* and *SUZ12*. Obviously, those two subgroups of patients experienced poor response to sunitinib treatment and shorter progression-free survival (PFS). Verbiest et al. (2018) reported the similar outcome in their study, which proved the resistance to pazopanib in ccrcc1/ccrcc4 subtypes.

In addition, lncRNA-SRLR overexpression is linked to sorafenib resistance through promotion of IL-6 transcription and activation of STAT3 (Xu et al., 2017). miR-942 is associated with sunitinib resistance by promoting the secretion of MMP9 and VEGF (Prior et al., 2014). miR-99a-3p, which targets ribonucleotide reductase regulatory subunit-M2 (RRM2), is downregulated in sunitinib-resistance RCC (Osako et al., 2019). Overexpression of breast cancer resistance protein BCRP/*ABCG2*, which is posttranscriptionally suppressed by miR-212-3p and miR-132-3p, is associated with superior response to sunitinib treatment in RCC patients (Reustle et al., 2018).

**Figure 1:**
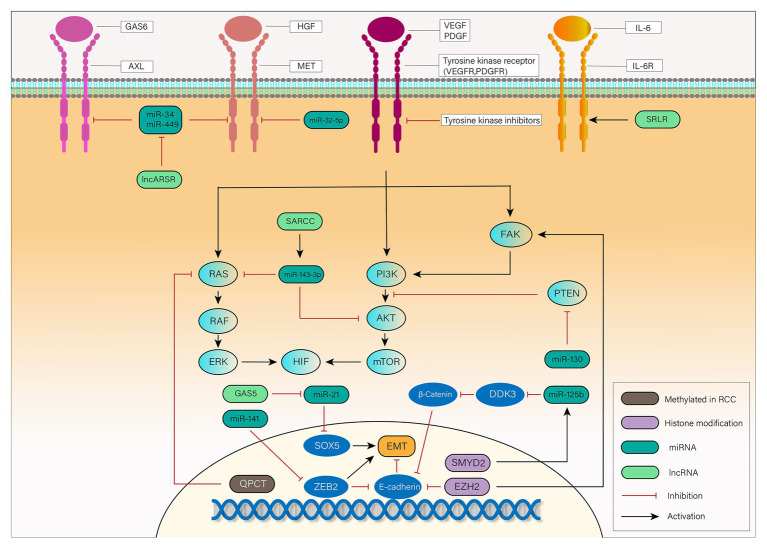
Mechanisms of tyrosine kinase inhibitor (TKI) resistance mediated by epigenetic alterations in renal cell carcinoma (RCC): vascular endothelial growth factor (VEGF) and platelet-derived growth factor (PDGF) bind to a tyrosine kinase receptor and activate the downstream focal adhesion kinase (FAK), PI3K, and RAS pathways. Activated FAK, PI3K, and RAS phosphorylate their downstream signaling cascade, eventually leading to the translation and accumulation of hydroxylated hypoxia inducible factor 1 (HIF-1α) and HIF-2α. In RCC, TKIs exert their influence on antiangiogenesis through inhibition of tyrosine kinase receptor. Epithelial-mesenchymal transition (EMT), activation of downstream signaling pathways and bypass pathways mediated by epigenetic alterations are responsible for the TKIs resistance. Long non-coding RNA (lncRNA)-SARCC increases miR-143-3p expression, thus inhibiting its downstream signals, including AKT, RAS, and ERK. miR-130 enhances HIF signaling by inhibition of PTEN. Hypermethylated *QPCT* reduces its protein level, leading to inhibition of RAS. EMT, a key transformation in TKIs resistance, is promoted by SOX5, zinc finger E-box binding 2 (ZEB2), and β-Catenin while inhibited by E-cadherin. lncRNA-GAS5 promotes EMT by competing with miR-21 which suppresses the expression of SOX5. miR-141 suppresses the expression of ZEB2 to inhibit its promotion of EMT. SET and MYND domain-containing protein 2 (SMYD2) leads to EMT by promoting the expression of miR-125b, which inhibits DDK3 and activates Wnt/β-catenin signaling pathway. The chromatin modifier enhancer of zeste homolog 2 (EZH2) can not only inhibit E-cadherin but also activate FAK signaling pathway to exert its influence on TKIs resistance. Except for VEGF receptor (VEGFR) and PDGF receptor (PDGFR), activation of MET, AXL, and IL-6 pathways can also lead to phosphorylation of downstream transduction cascades, such as PI3K, STAT3, and RAS. lncRNA Activated in RCC with Sunitinib Resistance (lncARSR) inhibits miR-34 and miR-449, and thus activates MET/AXL pathway. miR-32-5p inhibits MET pathway while lncRNA-SRLR activates interleukin-6 (IL-6)R pathway. RCC, renal cell carcinoma; VEGF, vascular endothelial growth factor; PDGF, platelet-derived growth factor; TKI, tyrosine kinase inhibitor; QPCT, the methylation of glutaminyl peptide cyclotransferase; AR, androgen receptor; EMT, epithelial-to-mesenchymal transition; EZH2, the chromatin modifier enhancer of zeste homolog 2; ZEB2, zinc finger E-box binding 2; SMYD2, SET and MYND domain-containing protein 2; GAS6, growth-arrest-specific protein 6; HGF, hepatocyte growth factor; IL-6, interleukin-6.

Some of the previously described epigenetic alterations associated with TKIs resistance are represented in Figure 1.

## Clinical Implications of Epigenetics Analysis in Rcc

Tyrosine kinase inhibitors treatment has been established as first-line therapy for mRCC for a decade with 70–80% of disease control rate. However, approximately 20–30% of patients does not respond to TKIs treatment and experience disease progression within ≤3 months (Porta et al., 2012). Epigenetic alteration can act as a biomarker, which predicts the response of patient to antiangiogenic therapy, thus reducing unnecessary toxicities and costs and maximizing clinical benefit. Clinical investigations of a number of epigenetic alterations on FFPE/plasma samples and their correlation with response to TKIs therapies are listed in Table 1.

**Table 1:** Epigenetic biomarkers in RCC patients treated with TKIs.

Classification	Epigenetic alteration	Study population	Sample source	TKIs treatment	Deregulation in TKI resistance	Reference
Histone	EZH2	16	tissue	sunitinib	↑	Adelaiye et al., 2015
DNA methylation	QPCT	10	tissue	sunitinib	↓	Zhao et al., 2019
	SYNPO2	63	tissue	Sunitinib, sorafenib, pazopanib	↓	Pompas-Veganzones et al., 2016
	NEFH	18	tissue	Sunitinib, sorafenib, axitinib, bevacizumab	↑	Dubrowinskaja et al., 2014
	CST6, LAD1	18	tissue	Sunitinib, sorafenib, axitinib, bevacizumab	↑	Peters et al., 2014
miRNA	miR-376b-3p	47	tissue	sunitinib	↓	Kovacova et al., 2019
	miR-9-5p	60	tissue	Sunitinib, pazopanib, sorafenib	↑	Ralla et al., 2018
	miR-489-3p	60	tissue	Sunitinib, pazopanib, sorafenib	↓	Ralla et al., 2018
	miR-628-5p	123	tissue	sunitinib	↓	Puente et al., 2017
	miR-27b	123	tissue	sunitinib	↓	Puente et al., 2017
	miR-99b-5p	40	tissue	Sunitinib, sorafenib, pazopanib	↓	Lukamowicz-Rajska et al., 2016
	miR-101	94	tissue	sunitinib	↓	Goto et al., 2016
	miR-155, miR-484	79	tissue	sunitinib	↑	Merhautova et al., 2015
	miR-942	20	tissue	sunitinib	↑	Prior et al., 2014
	miR-141, miR-144, miR-376b	20	tissue	sunitinib	↓	Berkers et al., 2013
	miR-520 g, miR-155, miR-526b,	20	tissue	sunitinib	↑	Berkers et al., 2013
	miR-424,	38	Plasma	sunitinib	↑	Gámez-Pozo et al., 2012
lncRNA	lncRNA-GAS5	15	tissue	sorafenib	↓	Liu et al., 2019
	lncRNA-SRLR	96	tissue	sorafenib	↑	Xu et al., 2017
	lncARSR	84	Plasma, tissue	sunitinib	↑	Qu et al., 2016

On the histone modification level, tissue low EZH2 expression was associated with increased overall survival (OS) in RCC treated with sunitinib (*p* = 0.005; Adelaiye-Ogala et al., 2017). On the DNA methylation level, tissue hypomethylation level in the CpG sites of *QPCT* promoter region showed a poor response to sunitinib therapy (*p* < 0.05; Zhao et al., 2019). Hypermethylation of *cystatin 6* (*CST6*), *ladinin 1* (*LAD1*) and *neurofilament heavy* (*NEFH*) were all linked to shortened PFS (*p* = 0.009, *p* = 0.011, and *p* < 0.001, respectively) and OS (*p* = 0.011, *p* = 0.043, and *p* = 0.028, respectively) for antiangiogenic therapy, including sunitinib, sorafenib, axitinib, and bevacizumab, among which methylation of *CST6* could predicted first-line therapy between response (0) and therapy failure (1) with an AUC of 0.88 and a sensitivity and specificity of 82 and 86%, respectively (Dubrowinskaja et al., 2014; Peters et al., 2014). Methylation level of *VHL* was found to be significantly upregulated after sunitinib therapy (*p* < 0.001; Stewart et al., 2016), while there was no correlation between *VHL* methylation and response to pazopanib (Choueiri et al., 2013). Beuselinck et al. (2015) reported the patients with tissue *MYC* overexpression and global CpG hypermethylation received a shorter PFS and OS after sunitinib treatment (*p* = 0.001 and *p* = 0.0003, respectively). In contrast, tissue unmethylation *SYNPO2*, the gene that encoded myopodin, discriminated progressing patients after TKIs treatment (sunitinib, sorafenib, and pazopanib) from those free of disease, and remained as an independent predictive factor for progression, disease-specific survival, and OS (*p* = 0.009, *p* = 0.006, and *p* = 0.01, respectively; Pompas-Veganzones et al., 2016).

On the ncRNA level, both miRNA and lncRNA showed their influences on the response to TKIs treatment. In their original study, Berkers et al. (2013) described the upregulation of miR-520 g, miR-155, and miR-526b and downregulation of miR-141, miR-376b in tissue were linked to the poor responders to sunitinib (*p* = 0.036, *p* = 0.04, *p* = 0.0067, *p* = 0.0098, and *p* = 0.032, respectively). In an observational prospective study, blood samples from 38 patients and 287 miRNAs were taken and evaluated before initiation of therapy and 14 days later in patients receiving sunitinib treatment for advanced RCC. Twenty eight miRNAs of the 287 were found to be significant differences of expressions between the poor response and response groups, among which, downexpression of miR-424 was linked with prolonged response (*p* = 0.016; Gámez-Pozo et al., 2012). Other researchers (Prior et al., 2014) explored a putative role of miRNAs in influencing sunitinib resistance to RCC in tissue, identifying that tissue overexpressed miR-942 was associated with sunitinib resistance, reduced time to progression (TTP) and OS (*p* = 0.0074, *p* = 0.003, and *p* = 0.0009, respectively), and predicted sunitinib efficacy with an AUC of 0.798 and a sensitivity and specificity of 92 and 50%, respectively. Lukamowicz-Rajska et al. (2016) reported that tissue decreased miR-99b-5p was associated with TKIs non-responders (sunitinib, sorafenib, and pazopanib) with a shorter PFS (<3 months, *p* < 0.0001). Similarly, Puente et al. (2017) identified that the expression of miR-23b, miR-27b, and miR-628-5p in tumor tissue was upregulated in long-term responders to sunitinib (*p* < 0.01, each), among which high level of miR-27b and miR-628-5p were associated with increased disease specific survival (*p* = 0.012 and *p* = 0.017, respectively). Nineteen miRNAs were explored to have different expressions in tissue, and lower level of miR-155 and miR-484 were associated with increased TTP in patients on sunitinib treatment (*p* < 0.01 and *p* < 0.05, respectively; Merhautova et al., 2015). Among 40 miRNAs of 232 found to be downregulated in sunitinib-treated RCC specimens compared with those in normal kidney tissues, miR-101 showed the most dramatic downregulation (*p* = 0.0013; Goto et al., 2016). Increased miR-9-5p and decreased miR-489-3p were found in non-responder patients of TKIs treatment, including sunitinib, sorafenib, and pazopanib compared to that in responder patients, and the AUC of miR-9-5p combined with clinicopathological variables to predict response(0)/non-response(1) to sunitinib treatment is 0.89 (Ralla et al., 2018). miR-212-3p and miR-132-30 were linked to shorter PFS of sunitinib therapy through interaction with BCRP/*ABCG2* expression (Reustle et al., 2018). In a more recent study, high-throughput miRNA microarray performed on FFPE tumor specimens from 47 patients treated with sunitinib, 158 miRNAs were identified to have different expressions in patient with good and poor response (*p* < 0.05). Moreover, miR-376b was significantly upregulated in patients with a long-term response to sunitinib and could identify patients with long-term response with a sensitivity of 83% and specificity of 67% (*p* = 0.0002, AUC = 0.758; Kovacova et al., 2019).

Regarding lncRNAs, relevant studies had disclosed their influences on TKIs therapy and prognosis. lncARSR was exposed in plasma and tissue separately by Qu et al. (2016), and was deemed to act as a sponge to compete with miR-34 and miR-449. High level of lncARSR in pre-surgery plasma was an independent prognostic factor for patients with sunitinib treatment, and was correlated with decreased PFS (*p* = 0.02 and *p* = 0.014, respectively). Intriguingly, low level of lncARSR in tissue exhibited a superior PFS after receiving sunitinib therapy (*p* = 0.028). A microarray analysis conducted by Xu et al. (2017) revealed the similar outcome in lncRNA-SRLR. Briefly, high tissue lncRNA-SRLR was associated with poor response to sorafenib, and patients with low lncRNA-SRLR expression had a more significant improvement in PFS after receiving sorafenib treatment (*p* = 0.0198 and *p* = 0.0086, respectively). lncRNA-GAS5 was also found to be downregulated in RCC patients with sorafenib resistance (*p* < 0.01; Liu et al., 2019).

Overall, these studies demonstrate that epigenetic alterations could be promising predictive biomarkers for TKIs response, as they function as important roles involved in mediating resistance through regulating important mechanisms. However, most of these studies are involved in a small number of patients, which limits their reliability. Moreover, there is no accepted criterion on how long a PFS of good response should last, so each study divides patients into good responders and poor responders based on its own standard. The different criteria limit the application of those epigenetic biomarkers in clinical setting.

## Epigenetic Alterations as Therapeutic Targets

Besides the predictive value, epigenetic alterations have potential to become targets themselves for drug development, in order to overcome the TKIs resistance in RCC. Preclinical studies on RCC cell lines demonstrate that reversions of epigenetic alterations are effective strategies to re-sensitize resistant clones to TKIs treatment, including demethylation, restoration of miRNA function, and inhibition of HDAC. Therefore, implementing epigenetics-based therapeutic strategies in patients is the next step, and relevant clinical trials are under way. Generally, there are two classes of epigenetics-based drugs in clinical trials: broad reprogrammers, which have a broad effect, and targeted therapies, which focus on specific miRNA expression or histone modifications (Jones et al., 2016). The formers are represented by the DNMT inhibitors (DNMTi) and the HDAC inhibitors (HDACi), and the latters are represented by EZH2 inhibitors (Jones et al., 2016; Joosten et al., 2018). The outcomes of current clinical trials concerning combination of epigenetics-based therapy with TKIs are listed in Table 2. So far, HDACi and EZH2 inhibitors are the most promising agents to reverse the TKI resistance with a vast of clinical studies completed or ongoing. Combination of HDACi and antiangiogenic agents is the most common trial to reverse the acquired resistance and re-sensitize tumors to antiangiogenic therapy. A phase I study evaluated the safety, tolerability, and preliminary efficacy of HDACi vorinostat plus sorafenib in patients with RCC and non-small cell lung cancer (NSCLC) and showed poor tolerance and no confirmed responses (Dasari et al., 2013). Other study focused on the combination vorinostat with pazopanib in advanced solid tumors including RCC, and identified that the treatment achieved stable disease for at least 6 months or partial response (PR; SD ≥ 6 months/PR) in 19% of all patients (*n* = 78), median PFS of 2.2 months, and median OS of 8.9 months (Fu et al., 2015). Furthermore, patients with detected hotspot *TP53* mutations had a superior rate of SD ≥ 6 months/PR, median PFS, and OS compared with those with undetected hotspot *TP53* mutations (45 vs. 16%, 3.5 vs. 2.0 months, and 12.7 vs. 7.4 months, respectively). In a phase I study, combination HDACi abexinostat with pazopanib in patient of RCC with tumor progression after received an average 2.5 lines of prior therapy and 1.6 lines of prior VEGF-targeting treatment received 27% of objective response rate and average 10.5 months of response duration (Aggarwal et al., 2017). Three patients with prior refractory disease to pazopanib monotherapy received durable minor or PR > 12 months treated with pazopanib plus abexinostat. Other clinical trial explored the effect of combination of HDACi with monoclonal antibody bevacizumab in advanced RCC. In a multicenter, single-arm phase I/II clinical trial, 33 patients with metastatic or unresectable ccRCC achieved 5.7 months of median PFS and 13.9 months of median OS, among which six patients achieved OR, including 1 CR and 5 PR (Pili et al., 2017). Regarding DNMTi, decitabine was the only agent tested by phase I trial and combination it with high-dose IL-2 achieved stable disease in three patients (Gollob et al., 2006). As the pharmacological defects of this DNMTi, such as short half-life and sensitivity to inactivation by cytidine deaminase, limit their clinical application, the second-generation DNMTi guadecitabine has been developed, which has shown promise in early preclinical models and clinical trial in patients with acute myeloid leukemia and myelodysplastic syndromes (Joosten et al., 2018).

**Table 2:** Epigenetic drugs plus TKIs in treatment of RCC.

Drug	Combination agent	Tumor type	Trial phase	Result	Reference
Vorinostat	Sorafenib	RCC, NSCLC	I	Poorly tolerated, 1 unconfirmed PR and five of eight patients had durable minor responses (11–26%) in RCC subset	Dasari et al., 2013
Vorinostat	Pazopanib	Solid tumors including RCC	I	Stable disease at least 6 months or PR (SD ≥ 6 months/PR), median PFS of 3.5 months and median OS of 12.7 months	Fu et al., 2015
Vorinostat	Bevacizumab	ccRCC	I/II	6 OR (18%), including 1 CR and 5 PR. 5.7 months of median PFS and 13.9 months of OS	Pili et al., 2017
Abexinostat	Pazopanib	Solid tumor including RCC	I	27% of objective response rate, average 10.5 months of response duration in RCC subset	Aggarwal et al., 2017

RCC, renal cell carcinoma; NSCLC, non-small cell lung cancer; ccRCC, clear cell renal cell carcinoma; PR, partial response; CR, complete response; OS, overall survival; PFS, progression-free survival; SD, stable disease.

The H3K27 histone N-methyltransferase EZH2 is a pusher of EMT leading to TKIs resistance, and its inhibitors may contribute to re-sensitize tumor to antiangiogenic treatment, which has been proven in preclinical test in RCC lines (Wagener et al., 2008; Adelaiye et al., 2015). The result of phase I trial that 64 patients including 21 with B-cell non-Hodgkin lymphoma and 43 with advanced solid tumors received EZH2 inhibitor tazemetostat showed the agent had a favorable safety profile and antitumor activity (Italiano et al., 2018).

Moreover, miRNAs also have the potential to become a target to reverse the TKIs resistance in RCC. Preclinical studies on RCC lines clearly demonstrated that both restoration of the tumor-suppressor miRNA function (by miRNA mimics) and inhibition of the oncogenic miRNAs (by antagomiRs) could re-sensitize resistance clones to TKIs. However, implementing miRNA-based therapies in clinic constitutes a significant challenge for clinicians and has not yet been realized. The main concerns fasten on the relative instability of miRNAs in body fluids and specific delivery of these miRNAs to tumor sites (Christopher et al., 2016; Leonetti et al., 2019). Recently, exosomes have been identified to function as carriers of miRNAs to deliver them from cell to extracellular milieu, which may become the sally port for miRNA-based therapy (Mathiyalagan and Sahoo, 2017; Rahbarghazi et al., 2019).

In addition, as epigenetic memory defines the ability of cells to retain and transmit their special gene expression status to the daughter cells, one differentiated somatic cell could become pluripotent and subsequently be reprogrammed into a different somatic cell through loss of its epigenetic memories responsible for its differentiated state. This process could serve as the basis for stem cell therapeutics by replacing one’s affected cells with his/her own cells, which may become the potential target of new agents (Thiagalingam, 2020).

Although agents targeting the epigenome could be a promising therapy strategy for TKIs resistance in mRCC because of the widespread epigenetic deregulation in this tumor type, there are several problems of those agents limiting their clinical application. For example, clinical activity of a drug is not only related to the original rationale but also attribute to the off-target effects. Studies about patients treated with epigenetic agents such as DNMTi revealed acute genome-wide demethylation (Yang et al., 2006), which may not only restore abnormally silenced expression but also activate normally silenced expression, leading to adverse off-targets effects. The individual responses of epigenetic agents are variable in different tumor types. So far, hypomethylating drugs are generally more effective in myeloid malignancy than in RCC. Furthermore, the majority of patients have been treated with DNMTi or HDACi for a shortened period of time, the long-term effects of those agents are not explicit for us. In addition, combination treatment might bring more severe and dose-limiting toxicities than monotherapy. As a result, additional trials are urged to future elaborate the interaction of those agents with mechanism of TKIs resistance and to assess their use in RCC patients.

## Conclusion

Although the advent of TKIs therapy indeed provides concrete hope for patients with advenced RCC, a part of patients with intrinsic or acquired resistance to TKIs benefit a little from the therapy and experience tumor progression after treatment. Epigenetic alterations are involved in the mechanisms underlying this event and could act as excellent biomakers to predict the response of patients to TKIs treatment. However, no epigenetic biomarker is currently applied in clinical setting regardless of numerous epigenetic biomarkers reported. Low efficiency and high cost of them may be the cause of this event. Therefore, for the purpose of translation them into clinical practice, more high-quality epigenetic biomarker studies are needed. In view, the criteria of TKIs resistance are ambiguous, uniform defination of TKIs resistance is urgent affair. Assay, statistical methods, and study designs also need be standardized to optimize their practice. In addition, epigenetics-based therapies are in full swing, which hold great promise and may optimize the management of patients with advanced RCC.

## Author Contributions

QL and ZZ searched the related published articles and wrote the manuscript. QZ and YF designed the work and instructed the progress of the review. All authors contributed to the article and approved the submitted version.

### Conflict of Interest

The authors declare that the research was conducted in the absence of any commercial or financial relationships that could be construed as a potential conflict of interest.
